# Color Stability of Enamel following Different Acid Etching and Color Exposure Times

**DOI:** 10.5681/joddd.2014.012

**Published:** 2014-06-11

**Authors:** Arezoo Jahanbin, Mohammad Basafa, Mostafa Moazzami, Behnoush Basafa, Neda Eslami

**Affiliations:** ^1^Associate Professor, Department of Orthodontics, Faculty of Dentistry, Mashhad University of Medical Sciences, Mashhad, Iran; ^2^Professor, Department of Orthodontics, Faculty of Dentistry, Mashhad University of Medical Sciences, Mashhad, Iran; ^3^Associate Professor, Department of Restorative Dentistry, Dental Research Center, Faculty of Dentistry, Mashhad University of Medical Sciences, Mashhad, Iran; ^4^Dentist, Private Practice, Mashhad, Iran; ^5^Assistant Professor, Department of Orthodontics, Dental Research Center, Faculty of Dentistry, Mashhad University of Medical Sciences, Mashhad, Iran

**Keywords:** Acid etching, staining, tooth discoloration

## Abstract

***Background and aims.*** The aim of this study was to evaluate the effect of different etching times on enamel color stability after immediate versus delayed exposure to colored artificial saliva (CAS).

***Materials and methods.*** Human first premolars were divided into five groups of twenty. A colorimeter was used according to the CIE system on the mid-buccal and mid-lingual surfaces to evaluate initial tooth color. Samples in group A remained unetched. In groups B to E, buccal and lingual surfaces were initially etched with phosphoric acid for 15 and 60 seconds, respectively. Then, the samples in groups A and C were immersed in colored artificial saliva (cola+saliva). In group B, the teeth were immersed in simple artificial saliva (AS). Samples in groups D and E were immersed in AS for 24 and 72 hours, respectively before being immersed in colored AS. The teeth were immersed for one month in each solution before color measurement. During the test period, the teeth were retrieved from the staining solution and stored in AS for five minutes. This was repeated 60 times. Color changes of buccal and lingual surfaces were calculated. Kruskal-Wallis and Wilcoxon tests were used for statistical analysis (α ≤0.05).

***Results.*** There were no significant differences between the groups in term of ΔE of buccal (P = 0.148) and lingual surfaces (P = 0.73).

*** Conclusion.*** Extended time of etching did not result in significant enamel color change. Immediate and delayed exposure of etched enamel to staining solutions did not result in clinically detectable tooth color changes.

## Introduction

One of the undesirable effects of bonding orthodontic attachments to enamel is the enamel color change at the end of treatment. Enamel color alterations can be divided into internal or external color changes.^1^ Pulpal bleeding or necrosis, meta-morphosis calcification, fluorosis, systemic drugs such as tetracycline and iatrogenic factors can cause internal color changes.^2-5^

External color alterations may occur due to cigarette smoking, staining drinks (such as coffee, tea and cola), ultraviolet irradiation and bonding of resin tag remnants to enamel.^6-8^These color changes are usually unsightly, especially in anterior areas and result in patient dissatisfaction with their orthodontic treatment.

Despite relatively numerous studies available on decalcification following acid etching, there is no evidence available on enamel color changes.^9^ Almost all of the previous studies concerning color stability have reported that different drinks can have various staining effects on composite resins depending on their composition and properties.^10-13^Ertas et al^14^reported that red wine caused more composite discoloration compared to water, cola, tea, and coffee. Similarly, Guler et al^15^ found that red wine produced the most severe discoloration in light-polymerized and microhybrid composite resins, followed by coffee and tea. In addition, Bagheri et al^16^ showed that coffee, tea and red wine caused more discoloration than soy sauce and cola. However, no study to our knowledge has studied staining effects of cola on etched enamel following immediate versus delayed exposure.

Although there are some studies on the effect of extended time of etching on bond strength, to the best of our knowledge, no study has evaluated the discoloration effect of enamel following extended etching time. Ito et al^17^found that a short etching duration (10s) provided higher bond strength than extended etching (30s, 60s) of samples contaminated with saliva and blood. Chousterman et al^18^recommended that extension of acid etching time to 90s will improve tensile bond strength of composite bonded to Er:YAG laser-prepared dentin. On the other hand, according to Oliveira et al^19^etching time had no significant effect on the bond strength of the adhesives to dentin. Contrary to this finding, Hiraishi et al^20^ found that extended etching time had an adverse effect on the microtensile bond strength for normal dentin.

Since tooth discoloration, especially after fixed orthodontic therapy, can cause dissatisfaction, the aim of this study was to evaluate the effect of different acid etching times on color stability of enamel following immediate versus delayed exposure to colored artificial saliva.

## Materials and Methods

One hundred freshly extracted human first premolars were selected for this study. The exclusion criteria included teeth with carious lesions, cracks, hypoplasia or obvious enamel discolorations. At first, all the crown surfaces were cleaned with pumice slurry.

Teeth were then mounted in 1 × 2 cm acrylic moulds 1.0 mm below the CEJ. Midpoints of the buccal and lingual surfaces were recorded using a clipper to allow repeated measurements of the same area. The centers of each buccal and lingual surface were evaluated by a colorimeter (Minolta CR-300, Minolta Co, Osaka, Japan) with the right angle according to the Commission Internationale del' Eclairage, L^*^, a^*^, b^*^ (CIE lab) system. In order to reduce the effect of external light, color measurements were made at midday in the same place every time.

The teeth were divided into five groups (n=20). In group A, the samples were not etched. In the remaining four groups (B-E), the buccal surface of each tooth was etched with 35% phosphoric acid (Ultra Etch, Ultradent Products Inc, USA) for 15 seconds, and then rinsed and dried for another 15 seconds. The same procedure was repeated for the lingual surface, except that it was etched for 60 seconds and washed so as not to cause critical contamination of the buccal surface.

In the next step, samples in groups A and C were immersed immediately in colored artificial saliva containing Nacl–Hydroxy Propyl Metyl cellulose and cola (water, sugar, caramel color, phosphoric acid, caffeine and favor) and in group B, the teeth were immersed only in artificial saliva (AS). Samples in groups D and E were immersed in AS for 24 and 72 hours, respectively, before being immersed in colored AS. All the samples were stored at 37°C.

To simulate clinical conditions as much as possible, the teeth were immersed for a test period of one month (2592000 seconds) in each solution before color measurement. Then color measurement was carried out with a colorimeter and color changes of the buccal and lingual surfaces were calculated. The colorimeter was calibrated according to manufacturers' instructions.

To reduce errors, colorimetric measurements were repeated with a one-hour interval in 50 samples and the mean values of L^*^, a^*^, b^*^ were calculated.

Statistical analysis was performed using SPSS 11.5. Differences between the groups were investigated using the non-parametric Kruskal-Wallis test. To compare color changes of the buccal and lingual surfaces in each group, the Wilcoxon test was used. Statistical significance was set at P < 0.05.

## Results

As shown in Table 1, Kruskal-Wallis test did not reveal a significant difference between the five groups in term of the ΔE of the buccal (P = 0.148) and lingual surfaces (P = 0.73). However, group B (P = 0.014) and E (P = 0.046) showed significant differences between the DE of the buccal and lingual surfaces.

**Table 1:** Means and standard deviations of color changes (ΔE) of the buccal and lingual surfaces of the test groups

Groups	Color change values (ΔE) mean ± SD	Color change values (ΔE) mean ± SD	Wilcoxon signed
	(Buccal surface)	(Lingual surface)	rank test
A	1.07 ± 0.12	1.31 ± 0.38	P = 0.093
B	1.45 ± 1.40	1.20 ± 0.53	P = 0.014^*^
C	1.07 ± 0.14	1.10 ± 0.25	P = 0.794
D	1.02 ± 0.01	1.07 ± 0.13	P = 0.296
E	0.07 ± 0.16	1.18 ± 0.18	P = 0.046^*^
Kruskal-Wallis test	P = 0.73	P = 0.148	
^ *^p <0.05			
Group A: The samples were exposed to staining solution without acid etching.			
Group B: After acid etching (15 seconds for buccal surface, 60 seconds for lingual surface), the samples were immersed in simple artificial saliva.			
Group C: After acid etching (15 seconds for buccal surface, 60 seconds for lingual surface), the samples were immersed in colored artificial saliva.			
Group D: After acid etching (15 seconds for buccal surface, 60 seconds for lingual surface), the samples were immersed in simple artificial saliva for 24 hours, and then exposed to colored artificial saliva.			
Group E: After acid etching (15 seconds for buccal surface, 60 seconds for lingual surface), the samples were immersed in simple artificial saliva for 72 hours, and then exposed to colored artificial saliva.			

## Discussion

Color changes in dentistry can be measured using spectrophotometers or colorimeters.^14^These instruments reduce subjective errors of color assessment with the naked eye.^16,21^CIELAB color system was developed by the Commission International d’Eclairage for measuring colors on the basis of human perception and it is widely used today for color assessment. DE (color difference value) shows the amount of color change in comparison to the baseline color.^23^

In this system, ΔE values greater than 3.7 units and ΔL values greater than 2 units are clinically unacceptable.^24,25^ΔL is more significant compared to Δa and Δb parameters because its changes can be detected more easily by the human eye. In the present study, ΔE of all the five groups were less than 3.7; therefore, these color changes were considered clinically imperceptible by the human eye.

The lighting condition under which color measurement is performed affects the colorimeter values.^13^Therefore, all the color evaluations were carried out at midday (9-10 a.m.). Furthermore, since evaluation of the color difference was the aim of the present study, the choice of illuminant was not considered important.

In the current study, we investigated the effect of extended time of etching on tooth color changes. There are clinical conditions in which we have to extend the standard etching time. For example, Firoozmand et al^27^reported that during bonding orthodontic brackets to bleached enamel, bond values lower than 10 MPa can be found when etching time is only 15 seconds. They found that acid etching for 30 seconds provided the highest bond strengths.

Based on the results of this study, extended time of etching up to 60 seconds caused more enamel discoloration in samples immersed in colored artificial saliva after 72 hours of etching (Group E, delayed exposure to colored agent), and also in samples immediately immersed in simple artificial saliva (Group B). However, it should be noted that the DE values of both groups were below 3.7 units and clinically undetectable. Further research with extended time of etching, before immersion in different solutions, is suggested to simulate clinical conditions.

In clinical conditions, the tooth labial surface is usually etched completely before bracket bonding. Although resin is applied to etched enamel, there remain some areas which are still uncovered. These areas are exposed to different colored agents and can cause unsightly enamel discoloration.

Since most of the young patients referred for orthodontic treatments drink cola during their meals, cola was used as a coloring agent to induce discoloration of etched enamel in the present study. The other reason for choosing cola as colored agent, beside the popularity, was its lower pH (2.8) in comparison with Fanta and Miranda, the other popular beverages in Iran.

According to Um and Ruyter,^11^ although Cola has a low pH and might damage the surface integrity of enamel, it does not produce as much discoloration as coffee or tea, which can be explained by its lack of yellow colorants. In this regard, Bagheri et al^16^ and Ertas et al^14^ reported more composite discoloration with coffee and tea than cola. Therefore, in clinical practice, patients should also be aware of staining effects of other drinks such as coffee or tea.

Almaaitah et al^27^evaluated the effect of fixed orthodontic appliances on tooth color in a prospective clinical study. They found that the average tooth color difference after orthodontic treatment was 2.85 units. Men and adolescents had greater color change than did girls and adults. In the present study, we used human first premolars that were extracted for orthodontic purposes. We did not have any information regarding the age or sex of patients.

It should be noted that in vitro studies may not be a reliable simulation of clinical situations and randomized clinical trials are necessary to establish these findings.

## Conclusion

Within the limitations of the present study, the following results have been drawn:

1. If the time of acid etching is limited to 15 seconds, it does not cause enamel staining following exposure to cola.
2. When time of etching was extended to 60 seconds, no significant color changes occurred after exposure to staining solutions.
3. Immediate and late exposure of etched enamel to colored artificial saliva did not result in a clinically detectable tooth color change.

## References

[1] Goldstein RE, Graber DA (1995). Complete Dental Bleaching, 2nd ed.

[2] Carvalho V, Jacomo DR, Campos V (2010). Frequency of intrusive luxation in deciduous teeth and its effects. Dent Traumatol.

[3] Maroto M, Barbería E, Planells P, García Godoy F (2005). Dentin bridge formation after mineral trioxide aggregate (MTA) pulpotomies in primary teeth. Am J Dent.

[4] Knösel M, Attin R, Becker K, Attin T (2008). A randomized CIE L*a*b* evaluation of external bleaching therapy effects on fluorotic enamel stains. Quintessence Int.

[5] Shin DH, Summitt JB (2002). The whitening effect of bleaching agents on tetracycline-stained rat teeth. Oper Dent.

[6] Mathias P, Rossi TA, Cavalcanti AN, Lima MJ, Fontes CM, Nogueira-Filho Gda R (2011). Cigarette smoke combined with staining beverages decreases luminosity and increases pigmentation in composite resin restorations. Compend Contin Educ Dent.

[7] Faltermeier A, Rosentritt M, Reicheneder C, Behr M (2008). Discoloration of orthodontic adhesives caused by food dyes and ultraviolet light. Eur J Orthod.

[8] Ogaard B, Rølla G, Arends J (1988). Orthodontic appliances and enamel demineralizationPart 1Lesion development. Am J Orthod Dentofacial Orthop.

[9] Hintz JK, Bradley TG, Eliades T (2001). Enamel color changes following whitening with 10 per cent carbamide peroxide: a comparison of orthodontically-bonded/debonded and untreated teeth. Eur J Orthod.

[10] Türkün LS, Türkün M (2004). Effect of bleaching and repolishing procedures on coffee and tea stain removal from three anterior composite veneering materials. J Esthet Restor Dent.

[11] Um CM, Ruyter IE (1991). Staining of resin-based veneering materials with coffee and tea. Quintessence Int.

[12] Borges AB, Marsilio AL, Pagani C, Rodrigues JR (2004). Surface roughness of packable composite resins polished with various systems. J Esthet Restor Dent.

[13] Guler AU, Yilmaz F, Kulunk T, Guler E, Kurt S (2005). Effects of different drinks on stainability of resin composite provisional restorative materials. J Prosthet Dent.

[14] Ertaş E, Güler AU, Yücel AC, Köprülü H, Güler E (2006). Stability of resin composites after immersion in different drinks. Dent Mater J.

[15] Güler AU, Yilmaz F, Yenisey M, Güler E, Ural C (2006). Effect of acid etching time and a self-etching adhesive on the shear bond strength of composite resin to porcelain. J Adhes Dent.

[16] Bagheri R, Burrow MF, Tyas M (2005). Influence of food-simulating solutions and surface finish on susceptibility to staining of aesthetic restorative materials. J Dent.

[17] Itoh T, Fukushima T, Inoue Y, Arita S, Miyazaki K (1999). Effect of water, saliva and blood contamination on bonding of metal brackets with a 4-META/MMA/TBB resin to etched enamel. Am J Dent.

[18] Chousterman M, Heysselaer D, Dridi SM, Bayet F, Misset B, Lamard L (2010). Effect of acid etching duration on tensile bond strength of composite resin bonded to erbium:yttrium-aluminium-garnet laser-prepared dentinePreliminary study. Lasers Med Sci.

[19] Oliveira GC, Oliveira GM, Ritter AV, Heymann HO, Swift EJ, Yamauchi M (2012). Influence of tooth age and etching time on the microtensile bond strengths of adhesive systems to dentin. J Adhes Dent.

[20] Hiraishi N, Yiu CK, King NM (2008). Effect of acid etching time on bond strength of an etch-and-rinse adhesive to primary tooth dentine affected by amelogenesis imperfecta. Int J Paediatr Dent.

[21] Joiner A (2004). Tooth colour: a review of the literature. J Dent.

[22] O’Brien WJ (2002). Dental Materials and their Selection, 3rd ed.

[23] Craig RG, Powers JM (2002). Restorative Dental Materials,11th ed.

[24] International Commission on Illumination. Colorimetry: Official recommendations of the international commission on illumination, 2nd ed. Bureau Central de la CIE: Vienna; 1986.

[25] Johnston WM, Kao EC (1989). Assessment of appearance match by visual observation and clinical colorimetry. J Dent Res.

[26] Firoozmand LM, Brandão JV, Fialho MP (2013). Influence of microhybrid resin and etching times on bleached enamel for the bonding of ceramic brackets. Braz Oral Res.

[27] Al Maaitah EF, Abu Omar AA, Al-Khateeb SN (2013). Effect of fixed orthodontic appliances bonded with different etching techniques on tooth color: a prospective clinical study. Am J Orthod Dentofacial Orthop.

